# Divergent germination strategies of *Phragmites australis* seeds for tidal flat gradient adaptation and the implications for coastal wetland restoration

**DOI:** 10.3389/fpls.2025.1598379

**Published:** 2025-07-29

**Authors:** Peng Jia, Dezhi Li, Caifen Yu, Jing Jia, Jiangtao Wang, Ying Wang, Jing Chen

**Affiliations:** ^1^ School of Ecological and Environmental Science, East China Normal University, Shanghai, China; ^2^ National Marine Environmental Monitoring Center, Dalian, China; ^3^ Shanghai Key Lab for Urban Ecological Processes and Eco-Restoration, Shanghai, China; ^4^ Institute of Eco-Chongming (IEC), Shanghai, China; ^5^ Technology Innovation Center for Land Spatial Eco-restoration in Metropolitan Area, Ministry of Natural Resources, Shanghai, China; ^6^ Department of Ecology and Resources Engineering, Hetao College, Bayannur, China

**Keywords:** germination strategy, soil properties, salinity, seed storage temperature, ecological restoration

## Abstract

*Phragmites australis*, capable of both sexual and asexual reproduction, widely distribute along the tidal flat gradient in the Chongming Dongtan wetlands, eastern China. This study investigates whether *P. australis* exhibits maternal effects by examining how seed germination strategies are influenced by habitat origin, soil properties, storage temperature, and salinity conditions. Seeds were collected from different tidal flat habitats, and their germination responses were tested under varying salinity levels (0–2.0%), storage temperatures (4 °C and room temperature), and soil conditions. Germination rates, speeds, index and potentials were measured, and structural equation modeling (SEM) was used to analyze the effects of environmental factors. These results showed that when the salinity was up to 1.5%, the seed germination rate was 1.3% to 7.7%. When treated with a salinity level of 2.0%, the seed germination rates of different tidal flat populations decreased by 94.3% (L), 96.5% (IN), 100% (M) and 99.2% (H), respectively, compared to the control group. With the increase in salinity, the germination speeds of different tidal flats slowed down, and the germination index decreased. 4°C storage significantly enhanced the seed germination rate and germination potential of the low-tidal flat population (L) relative to room-temperature storage. Soil salinity and water content were major factors influenced germination rates after storing seeds at 4°C. However, germination rates and potentials were positively correlated with the soil phosphorus. Meanwhile, the seed germination indexes and speeds were more significantly affected by the room temperature. The SEM model explained 74% and 50% of the seed germination parameters under the seed storage condition at 4°C and room temperature, respectively. And the tide level of its directly decreasing effect for the seed germination parameters was higher at 4°C with -0.97 than at room temperature with -0.16. The results indicated that after ripening (low-temperature seed storage) differentially promoted the *P. australis* seed germination collected from the tidal flat gradient in a subtropical marine monsoon climate salt marsh and demonstrated maternal effect adaptation. Consequently, using seeds of *P. australis* along the tidal flat gradient to restore the original population could be considered an effective and economical method.

## Introduction

Loss and degradation, as well as the structural and functional damage of the coastal salt marsh wetlands, have become serious problems worldwide for centuries, directly or indirectly due to various human activities (e.g., dam construction) and/or global climate change (Kirwan and Megonigal, 2013; Mariotti and Fagherazzi, 2013). The coastal wetland destruction negatively impacts the environment, causing a decline in plant diversity and soil water content (Feng et al., 2022; Portnoy, 1999; Tan et al., 2020). This could potentially reduce plant resource utilization efficiency, soil organic matter, and the distribution of some native species while increasing the invasion of alien species (Warren et al., 2002; Portnoy, 1999; Portnoy and Giblin, 1997).

Some native plant species serve as foundation species in various ecosystems, maintaining their stability and functionality (Osland et al., 2014; Angelini et al., 2011). The loss of these native species could lead to damage or even collapse of the ecosystem. When restoring the degraded ecosystem, native foundation species often receive significant attention (Dupin et al., 2022). Therefore, it is essential to examine the ecological characteristics of native foundation plant species in various ecosystems. It may provide a crucial theoretical foundation and technical support for implementing effective ecological restoration practices in damaged ecosystems.

*P. australis* is one of the important native foundation plant species in the coastal wetland in eastern China (Suir et al., 2022; Wang et al., 2010), acting as a primary producer and the carbon sink (Stankovic et al., 2021). *P. australis* dominates in terms of biomass in numerous wetlands (Eller et al., 2020), particularly in China, and has been employed in various ecological restoration initiatives in damaged wetland areas (Sun et al., 2015; Wang et al., 2012; Xu et al., 1999). *P. australis* efficiently colonizes bare areas via vegetative propagation, and its seeds are effectively dispersed by water and wind, resulting in a more rapid expansion of the population area compared to growth solely through rhizomes (Eller et al., 2017; Kühl and Zemlin, 2000). In Europe, it was found that both sexual and asexual reproduction contributed to the fast dispersal of *P. australis* population, and during the colonization stage, it primarily began from seed germination (Koppitz and Kuehl, 2000). Although seed restoration is a low-cost and high-efficiency method for rehabilitating degraded salt marsh wetland vegetation, the seed germination process is often affected by various factors, including hydrological conditions, soil salinity, temperature and so on (Haraguchi, 2014). Seed germination is highly sensitive to the immediate environment of the seed as well as the environment experienced by the mother plant during seed development (Baskin et al., 2003). The maternal environment can affect the dormancy intensity of seeds, and thus affect the germination traits (such as germination rate and germination speed, etc.) (Song et al., 2016; Auge et al., 2017; Bhatt et al., 2020). It is well known that different populations can exhibit local adaptation in response to spatial variability in environmental conditions such as climate, hydrology, and soil parameters (Elnaggar et al., 2019; Bhatt et al., 2020; Leimu et al., 2008). The salinity of coastal salt marshes is considered to be one of the important factors affecting seed germination (Fernandez-Torquemada and Sanchez-Lizaso, 2013; Greenwood and MacFarlane, 2006; Xiao et al., 2016), so a better understanding of the effect of salinity on the mother plant could result in more saline-tolerant seeds, thereby accelerating population colonization and helping to identify suitable habitats for future restoration projects. Therefore, reversing the global salt marsh decline through restoration requires a comprehensive understanding of how different environmental factors impact the colonization and expansion of tidal marsh species, especially the important native foundation plant species (He and Silliman, 2019; Silliman et al., 2015).

Dongtan, as an internationally important wetland for bird migration in Eurasia, provides abundant food, shelter and habitats (Yuan et al., 2020). In Chongming Island, a large scale of *P. australis* populations were distributed in the middle and high tidal flats of Dongtan wetland, but they can also be found at low tidal flats with high soil salinity near the coastline, in the form of scattered patches, showing its adaptation to various coastal environmental conditions. Based on the wide ecological adaptability of *P. australis* in coastal wetlands, this study proposed the following hypotheses: (i) seeds of *P. australis* populations along the tidal flat gradient of the Dongtan salt marsh wetland will show divergent germination strategies, which will be influenced by the long term adaptation of the original populations to the local habitats, especially the soil physicochemical properties; (ii) soil salinity as a main environmental factor along the tidal flat gradient will seriously influence the germination of seeds of *P. australis* populations, and the germination strategies of seeds collected from different tidal flats will correspond to the soil salinity of the original habitats; (iii) in the subtropical marine monsoon climate area, *P. australis* seed germination will also be improved by the lower seed storage temperature as an often necessary condition for seed break dormancy.

## Materials and methods

### Study area

Dongtan as a coastal salt marsh wetland locates in the Chongming Island at the estuary of Yangtze River (121°50′E-122°5′E, 31°25′N-31°38′N). The area locates in the northern edge of the mid-subtropical zone with the marine monsoon climate. The average annual temperature is 15.3°C and the annual precipitation is 1100 mm (Jia et al., 2022).

The seeds of *P. australis* populations were collected from the Dongtan wetland at a tidal estuary with an irregular semi-diurnal tide type, and the daily tidal flat characterized by two tidal changes of day and night. According to the observations in recent decades, the mean sea level is 2.17 m, the mean low water level is 1.03 m, and the mean high water level is 3.5 m (Yuan et al., 2020). The area is densely covered with tidal creeks, and the zones of high, middle and low tidal flats are very obvious. Along the elevational gradient of the transect, three tide sampling locations were established including the high tidal flat (2.7–3.5 m), the middle tidal flat (2.4–2.7 m), and the low tidal flat (2.2–2.4 m). The *P. australis* populations at the middle and high tidal flats in the Dongtan marsh wetland were distributed in continuous bands, while at the low tidal flat, it was distributed in patches.

### Experimental design

#### Seed collection and storage

Given that *P. australis* is polyploid, with ploidy levels ranging from 4x to 12x, non-coding chloroplast DNA (cpDNA) has been the marker of choice in many studies due to its typical maternal inheritance (Saltonstall, 2001, 2016). Researchers have shown that haplotype P, representing an octoploid lineage, is now extensively dispersed in eastern China, with a concentration in coastal salt marshes (Liu et al., 2023, 2022). In late October 2022, seeds of *P. australis* were collected from the populations along the high, middle, and low tidal flats of the Dongtan salt marsh. Seed maturity and size were designed completely randomly in this study, and collected seeds were stored at 4°C and 20 ± 1 °C (room temperature) until the experiment began (the storage period is from early December 2022 to mid-March 2023). The seed storage temperatures simulated the typical average temperatures in the natural habitats. There was no significant difference in 100-grain weight among different habitats (**Supplementary Figure S1**).

#### Seed germination experiments

Seeds were taken out after being stored in the incubator, and then the palea and lemma were peeled from the seeds separately. Firstly, seeds were soaked in 2% sodium hypochlorite solution which was used to disinfect for 10 min, constantly stirring to ensure full contact between the seeds and the solution Secondly, seeds were washed repeatedly with pure water for 4 times to remove the residual disinfectant on them. Finally, they were soaked in the distilled water for 24 h. The seeds were cleaned and disinfected and placed evenly in Petri dishes (Φ= 9 cm), containing 2 mL of NaCl solution with salinity of 0%, 0.5%, 1%, 1.5% and 2%, respectively. Such salinities simulated the typical soil salinities (Biemond et al., 2023; Cai et al., 2015), and the extreme salty tides invading the natural habitats of *P. australis* populations. The evaporated water from the Petri dishes was replenished every day according to the calibrations.

Each salinity treatment consisted of 3 replicates; and each replicate contained 50 seeds. Seed germination experiments were carried out in an incubator with alternating light and dark conditions at 25/16°C for 14/10 h light/dark regime for 21 d. The standard for identifying seed germination was that the radicle penetrating the episperm and the bud length reaching to 1 mm (Yu et al., 2012). The situations and the numbers of germinated seeds were observed and recorded daily for 21 days, and the parameters (such as germination rate (*Gt* (Equation 1)) (%), germination index (*GI* (Equation 2)), germination speed (*Gs* (Equation 3)) and germination potential (Equation 4) (%)) were calculated.


(1) Gt=final actual number of germinated seeds in each Petri dish/number of tested seeds×100%

(2) GI=∑Gt/Dt

(3) $$G_{s}=[N_{1}+(N_{2}-N_{1})/2+(N_{3}-N_{2})/3+\ldots +(N_{n}-N_{n-1})/n]\times 100$$

(4) Germination potential=cumulative number of germinated seeds in the first 4 days/number of tested seeds×100%

Where the *Gt* is the number of germinated seeds at day t (7 d); Dt is the number of days. *G_s_* is the speed of germination; *N_n_* is the total number of days for observing the germinated seeds.

Germination pattern of seeds, which were collected from the populations in the original habitats and then they were treated under different seed storage temperatures (4°C as lower temperature and 20 ± 1°C as room temperature, respectively), were investigated, including a total of 24 Petri dishes (4 types of habitats × 3 replicates for each type of habitat × 2 storage temperatures). The numbers of germinated seeds were recorded daily for 21 days, and the above germination traits were calculated.

#### Collection of soil samples and measurement of physicochemical properties

**Figure 1:**
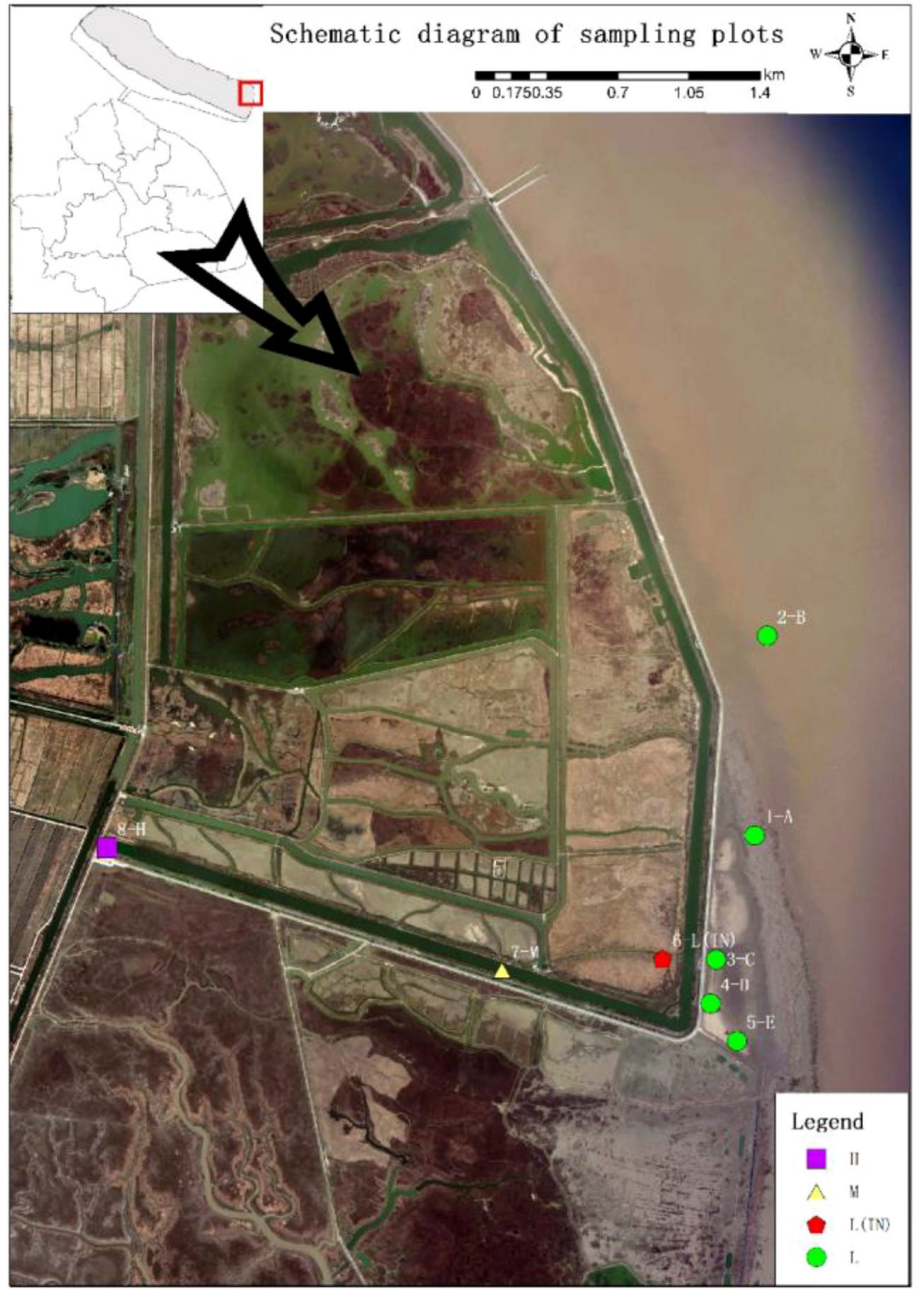
Study area and sample site location (L: low tidal flat outside the dyke; L(IN), low tidal flat inside the dyke; M, middle tidal flat; H, high tidal flat).

Sampling plots were set up in *P. australis* populations at different tidal flats in the Dongtan salt marsh wetland. Taking the dykes built in 1998 and 2002 as the boundary, respectively, the plots near the coast were named as L group and those within the dyke were named as IN group. At the low tidal flat outside the dyke, 5 patches with a distance of 500 m were randomly set and was named as A, B, C, D and E, respectively. A 15 m × 15 m plot was set in each patch, and then, three 1 m × 1 m sampling quadrats were randomly set in the plot. In the sampling plots within the dyke (IN), five 1 m × 1 m sampling quadrats were randomly set with a distance of 50 m. At the middle (M) and high tidal flats (H), where *P. australis* populations distributed in large areas, ten 1 m × 1 m quadrats were set with 20 m apart at each tidal flat (**Figure 1**). Three soil column samples (Φ = 5 cm, *H* = 20 cm) were randomly taken from each plot; each soil column sample was divided into two depths (0–10 cm, 10–20 cm). Soil samples at each depth were mixed, and then they were put into different cotton cloth bags brought back to laboratory for measuring the physicochemical properties, such as salinity, pH, water content, total nitrogen (TN), total phosphorus (TP), available phosphorus (AVIP), nitrate nitrogen (NO_3_^–^N), ammonium nitrogen (NH_4_^+^-N) and organic carbon (SOC) were detected according to the other method (Feng et al., 2022; Jia et al., 2022). Soil microbial biomass C (MBC) and microbial biomass N (MBN) were measured by an improved fumigation method (Feng et al., 2022).

### Data analysis

SPSS 22.0 was used for analyzing and comparing the data (mean ± standard error) and the significance test. In the absence of interaction, data from the germinated seeds was pooled, and the effects of salinity on the germination parameters were analyzed using one-way ANOVA and the Tukey test for the least significant difference (*p*=0.05). Two-way ANOVA was used to analyze the interaction of tidal level and treatment salinity on seed germination. Redundancy analysis (RDA) was used to analyze the differences in the correlation between the germination parameters of seeds stored under different temperatures and the soil physicochemical factors (pH, water content, salinity, TP, TN and SOC). Structural equation modeling (SEM) was used to evaluate and quantify the effects of soil physicochemical factors on the differences of germination parameters of seeds collected from the populations at different tidal flats and stored under different temperatures. RDA analysis and SEM used the “vegan” package (Dixon, 2003) and “lavvan” package (Rosseel, 2012) in R 4.1.2 version, respectively.

## Results

### Effects of soil salinity on seed germination

**Figure 2:**
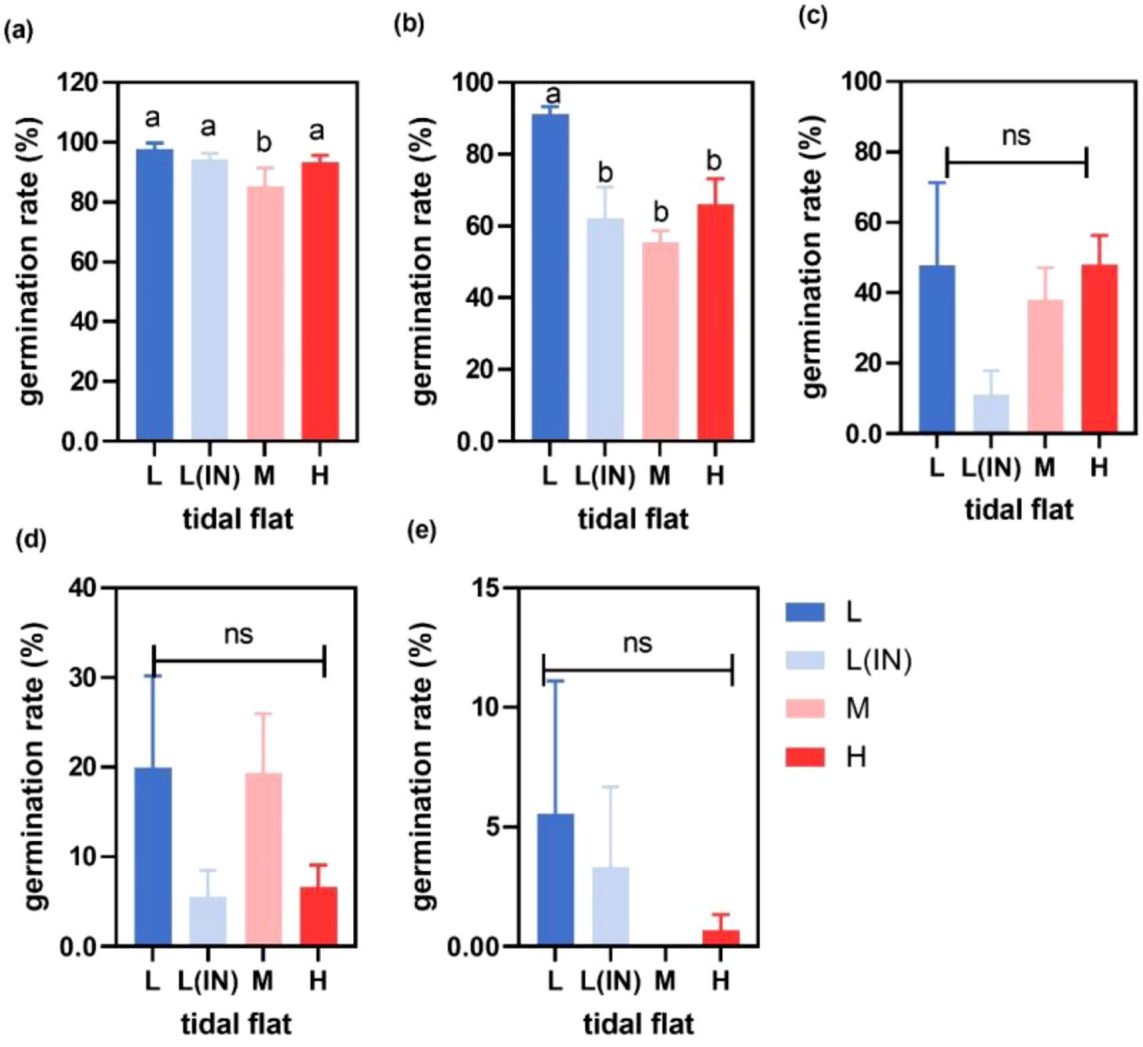
Germination of *P. australis* seeds collected from the populations at different tidal flats and treated with different salinities conditions **(a)** water treatment, **(b)** 0.5% salinity treatment, **(c)** 1% salinity treatment, **(d)** 1.5% salinity treatment, **(e)** 2% salinity treatment. (L, low tidal flat outside the dyke; L(IN), low tidal flat inside the dyke; M, middle tidal flat; H, high tidal flat. Different letters indicated significant differences between treatments and germination parameter, p<0.05, Error bars represented ± SD.).

With the increase of soil salinity, the germination rates, germination indexes and germination speeds of *P. australis* seeds collected from populations at different tidal flats decreased compared with the control group at 0% salinity (**Figures 2a–e**; **Supplementary Tables S1**, **S2**). When the treatment salinity was 0.5%, the seed germination rate of the *P. australis* population in the L tidal flat (91.1% ± 0.38) was significantly higher than that in the other several tidal flats(L(IN): 62.2% ± 1.5; M: 55.3% ± 0.5 and H: 66% ± 1.2). At 1% and 1.5% salinity, *P. australis* seeds collected from the populations at all tidal flats germinated, and the seed germination rates among the tidal flats showed no significant difference, but the germination rate of seeds collected from the populations at the L tidal flat outside the dike was higher than those of seeds collected from populations at the other sites (**Figures 2c, d**). At 2% salinity, only the seeds collected from the populations at the low tidal flat germinated, and the germination rates decreased by 90% compared with the control group (**Figure 2b**). The mixed linear model showed that soil salinity, tidal flat and their interactions affected the seeds germination, and the salinity was the main factor (**Table 1**).

**Table 1:** Results of two-way ANOVAs testing the effects of salinity, the tidal flats and their interactions on the germination indexes of *P. australis* seeds collected from the populations at the low, middle and high tidal flats in the Dongtan wetland.

Variables	Tidal flats				*F*		
	L	L(IN)	M	H	S	T	S×T
*Gt*	0.63 ± 0.03a	0.42 ± 0.02b	0.63 ± 0.03a	0.48 ± 0.04b	223.348***	50.707***	9.557***
*Gs*	10.49 ± 1.69a	3.18 ± 0.63c	7.36 ± 0.83b	1.49 ± 0.25c	112.515***	74.092***	18.867***
*GI*	0.51 ± 0.20a	0.15 ± 0.02b	0.39 ± 0.15a	0.10 ± 0.03b	30.731***	23.191***	4.075***
*GP*	0.52 ± 0.13a	0.08 ± 0.02c	0.31 ± 0.04b	0.07 ± 0.02c	38.805***	29.446***	10.561***

*Gt*, germination rate; *Gs*, germination speed; *GI*, germination index; GP, germination potential; L, low tidal flat outside the dyke; L(IN), low tidal flat inside the dyke; M, middle tidal flat; H, high tidal flat; S, Salinity; T, tidal flat. The contrasting lowercase letters indicated the significant differences among the different tidal flats. ***: *p <* 0.001.

### Effects of storage temperatures on seed germination

**Figure 3:**
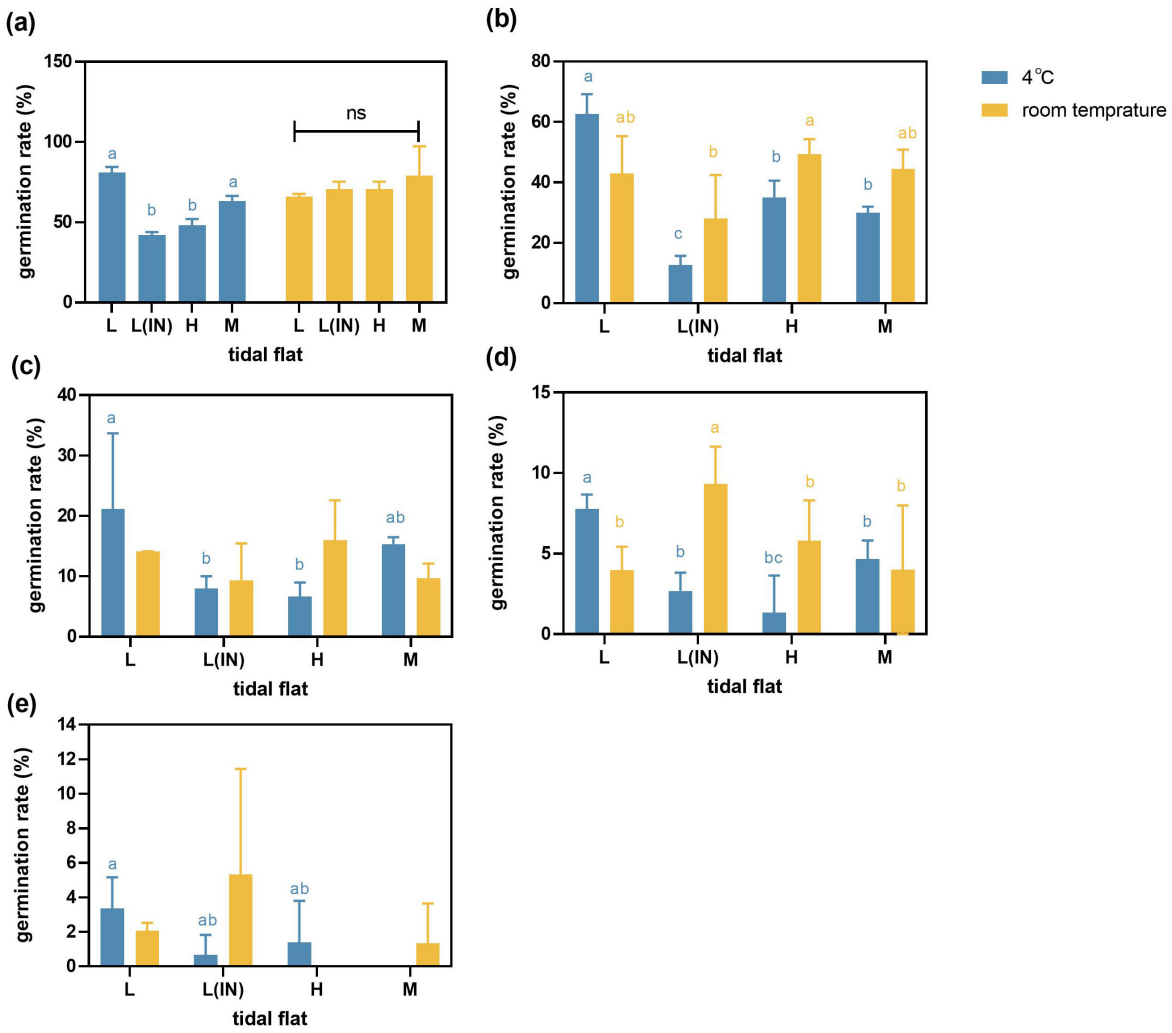
Germination of *P. australis* seeds collected from the populations at different tidal flats and treated with different salinities under the two temperature storage conditions. (L, low tidal flat outside the dyke; L(IN), low tidal flat inside the dyke; M, middle tidal flat; H, high tidal flat. **(a)** water treatment, **(b)** 0.5% salinity treatment, **(c)** 1% salinity treatment, **(d)** 1.5% salinity treatment, **(e)** 2% salinity treatment. Error bars represented ± SD.). The contrasting lowercase letters (a, b, c) indicated the significant differences among the different tidal flats.

The germination rates of *P. australis* seeds collected from populations at the high, middle and low tidal flats inside the dike, and stored at the room temperature (20 ± 1°C) decreased compared to those stored at the low temperature (4°C). The germination rate of seeds collected from the populations at the L tidal flat and stored at the room temperature decreased by 8%-12% compared to those stored at 4°C condition (**Figures 3a–d**), but the germination rates of seeds were basically kept the same when the treatment salinity was 2% (**Figure 3**). When the treatment salinity was 0.5%, the seed germination rates of the *P. australis* populations in the L, L(IN), M and H tidal flats under room temperature storage were 43.0% ± 12.23, 28.0% ± 14.42, 44.4% ± 6.36 and 49.2% ± 5.02, respectively. However, under the same treatment salinity, the seed germination rates of the *P. australis* populations in the L, L(IN), M and H tidal flats under low-temperature storage were 62.6% ± 6.63, 12.7% ± 3.05, 30.0% ± 2.0 and 35.05% ± 5.47, respectively. When the salinity was treated at 1%, 1.5% and 2% respectively, the seed germination rates of L(IN), M and H tidal flats were increased under room temperature storage conditions compared with low-temperature storage conditions (**Figures 3b–e**). Regardless of the treatment salinity conditions, the germination rate of L tidal flat seeds after low-temperature storage was higher than that of seeds under room temperature storage conditions (**Supplementary Tables S1**, **S2**).

### Soil physicochemical properties in the original habitats of *P. australis* populations at different tidal flats

Total nitrogen (TN), organic carbon content (Organic C), pH and available phosphorus (AVIP) of different soil layers in the original habitats of *P. australis* populations were significantly different. The contents of NO_3_^–^N (0.18 ± 0.07 mg/kg), NH_4_^+^-N (1.02 ± 0.17 mg/kg) and TN (0.1 ± 0.05g/kg) show a trend that they were higher at high tidal flat than at middle (NO_3_^–^N (0.20 ± 0.07 mg/kg), NH_4_^+^-N (0.91 ± 0.04 mg/kg) and TN (0.05 ± 0.02g/kg)) and low tidal flats (NO_3_^–^N (0.17 ± 0.04 mg/kg), NH_4_^+^-N (0.96 ± 0.17 mg/kg) and TN (0.09 ± 0.02g/kg)). Soil physicochemical indexes of different tidal flats were all significantly different, except for total phosphorus (TP). 0–10 cm soil layer salinity at the low tidal flat (0.5 ± 0.18 PSU) was higher than that at the middle (0.33 ± 0.08 PSU) and high tidal flats (0.29 ± 0.14 PSU), respectively, and soil salinity at 0–10 cm layer was higher than that at 10–20 cm layer. The soil pH of the low-tidal flat (8.12 ± 0.24) and the middle-tidal flat (8.31 ± 0.39) were higher than that of the high-tidal flat (7.92 ± 0.38), while the soil salinity of the 0–10 cm layer was lower than that of the 10–20 cm layer. This phenomenon indicates that the soil gradually tends to become salinized from the high tidal flat to the low tidal flat. The soil available phosphorus (AVIP)content in the tidal flat was the highest, being 21.15 ± 3.55 mg/kg. The variation trends of soil water content and AVIP were consistent with those of soil salinity at the tidal flats, but were opposite with those of soil salinity between soil layers. There was no interaction between soil physicochemical properties of soil layers and tidal flats (**Table 2**).

**Table 2:** Results of one-way ANOVAs testing the effects of soil depth at different tidal flats on the soil physicochemical properties of the habitats of *P. australis* populations, and two-way ANOVAs testing the effects of soil depth and different tidal flats on the soil physicochemical properties of the habitats of *P. australis* populations.

Variables	Soil depth	Tidal flat				*F*		
		L	IN	M	H	Soil depth	Tidal flat	Soil depth*Tidal flat
Salinity (PSU)	0-10cm	0.50 ± 0.18a	0.35 ± 0.13a	0.33 ± 0.08a	0.29 ± 0.14ab	0.427ns	14.155***	0.33ns
	10-20cm	0.47 ± 0.10a	0.35 ± 0.10a	0.34 ± 0.14a	0.22 ± 0.08ab			
NH_4_^+^-N(mg/kg)	0-10cm	0.96 ± 0.17	0.97 ± 0.05	0.91 ± 0.04	1.02 ± 0.17	0.025ns	2.413***	0.479ns
	10-20cm	0.91 ± 0.24	1.04 ± 0.09	0.88 ± 0.04	1.00 ± 0.08			
NO_3_^–^N(mg/kg)	0-10cm	0.17 ± 0.04b	0.47 ± 0.58a	0.20 ± 0.07b	0.18 ± 0.07b	0.63ns	6.826*	0.605ns
	10-20cm	0.16 ± 0.05b	0.35 ± 0.12a	0.17 ± 0.05b	0.22 ± 0.11b			
TN (%)	0-10cm	0.09 ± 0.02ab	0.06 ± 0.02ab	0.05 ± 0.02bc	0.10 ± 0.05a	4.022*	10.412***	0.046ns
	10-20cm	0.07 ± 0.02a	0.05 ± 0.01a	0.04 ± 0.01ab	0.08 ± 0.05a			
TP(g/kg)	0-10cm	0.07 ± 0.00	0.08 ± 0.00	0.07 ± 0.00ab	0.07 ± 0.01	1.697ns	2.11ns	0.233ns
	10-20cm	0.07 ± 0.00	0.07 ± 0.00	0.07 ± 0.00	0.06 ± 0.01			
Organic C (%)	0-10cm	0.85 ± 0.14 ab	0.60 ± 0.21 ab	0.52 ± 0.16ab	1.04 ± 0.65a	5.081*	9.888***	0.059ns
	10-20cm	0.68 ± 0.13a	0.41 ± 0.11ab	0.40 ± 0.10ab	0.85 ± 0.48a			
Moisture (%)	0-10cm	0.54 ± 0.18	0.34 ± 0.09	0.32 ± 0.09	0.51 ± 0.22	1.301ns	5.636***	0.931ns
	10-20cm	0.45 ± 0.14	0.30 ± 0.01	0.37 ± 0.15	0.43 ± 0.17			
pH	0-10cm	8.12 ± 0.24a	7.73 ± 0.30ab	8.31 ± 0.39a	7.92 ± 0.38a	16.103***	13.9***	0.484ns
	10-20cm	8.32 ± 0.21b	8.04 ± 0.17b	8.70 ± 0.18a	8.14 ± 0.36b			
AVIP (mg/kg)	0-10cm	21.15 ± 3.55a	20.18 ± 3.71a	8.98 ± 4.19b	13.56 ± 7.74b	4.617*	27.231***	0.481ns
	10-20cm	19.33 ± 4.43a	14.78 ± 4.01ab	7.18 ± 1.86bc	12.52 ± 6.60ab			
MBC (mg/kg)	0-10cm	2.95 ± 1.20a	1.18 ± 0.60ab	1.94 ± 1.18a	3.31 ± 2.62a	0.027ns	4.065**	0.531ns
	10-20cm	2.77 ± 0.93	2.19 ± 0.78	1.73 ± 1.25	2.94 ± 1.99			
MBN (mg/kg)	0-10cm	0.06 ± 0.03	0.08 ± 0.06	0.05 ± 0.04	0.14 ± 0.11	0.148ns	6.739***	0.652ns
	10-20cm	0.06 ± 0.04	0.13 ± 0.09	0.05 ± 0.05	0.12 ± 0.09			

TN, total nitrogen content; TP, total phosphorus content; Organic C, total organic carbon content; AVIP, available phosphorus content; MBC, microbial carbon content; MBN, microbial nitrogen content. The content values in the table were expressed as Mean ± SD. The contrasting lowercase letters indicated the significant differences among the different tidal flats; ns, no significant; ***p < 0.001; **p < 0.01; *p < 0.05.

### Correlation between seed germination parameters under different seed storage conditions and soil physicochemical factors of original habitats of *P. australis* populations

**Figure 4:**
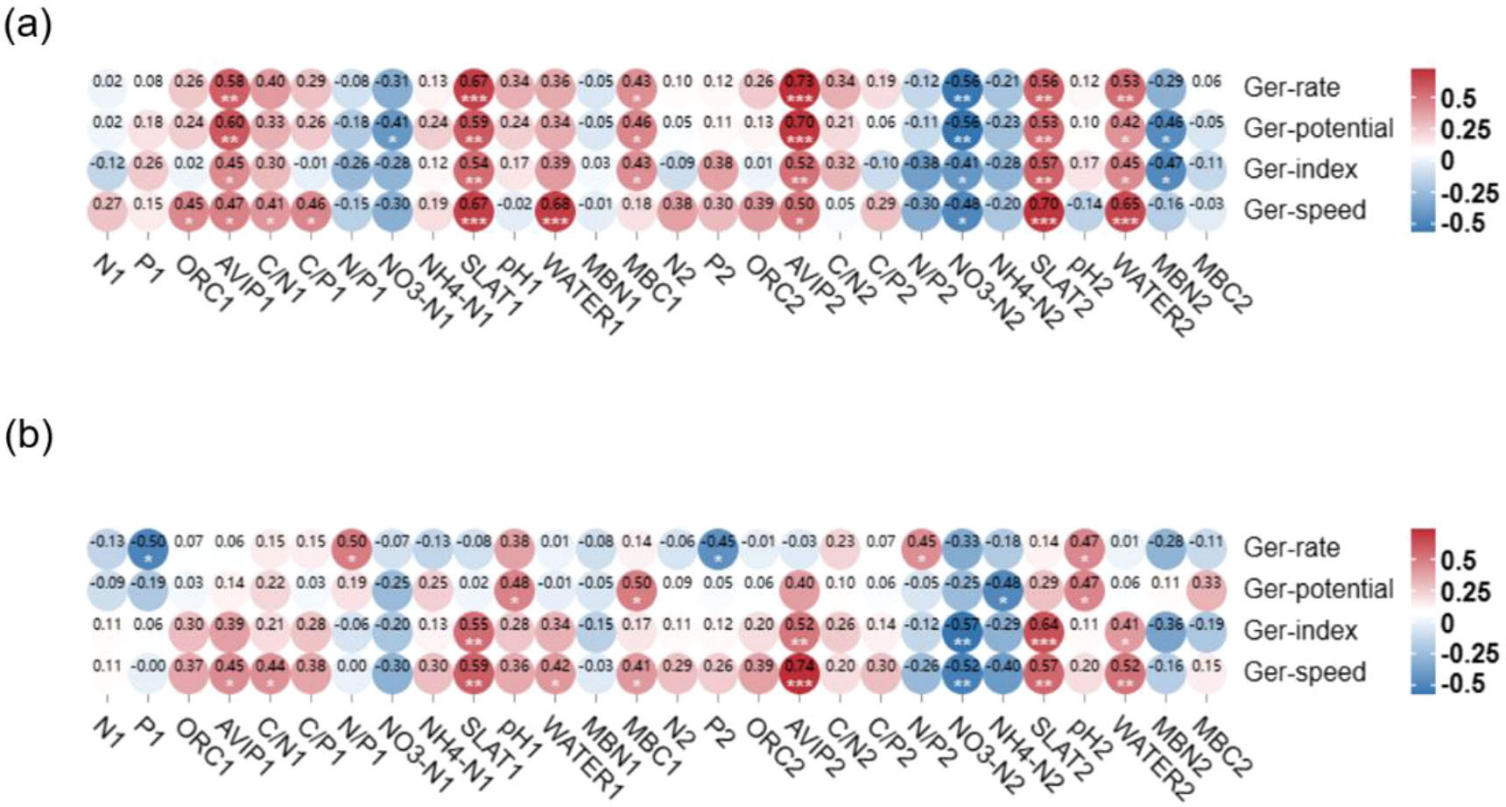
Correlation between the seed germination parameters of untreated seeds subjected to two storage temperatures and the soil physicochemical properties of their original habitat populations. **(a)** 4°C storage condition, **(b)** room temperature storage condition. (Ger-rate, germination rate; Ger-potential, germination potential; Ger-index, germination index; Ger-speed, germination speed; TN, total nitrogen content; TP, total phosphorus content; ORC, total organic carbon content; AVIP, available phosphorus content; MBC, microbial carbon content; MBN, microbial nitrogen content; 1 and 2 represent different soil layers).

The correlation analysis between seed germination parameters under 4 °C seed storage condition and soil physicochemical factors of original habitats of *P. australis* populations showed that germination rates, germination potentials, germination indexes and germination speeds were significant positively correlated with soil salinity, AVIP and water content of original habitats of *P. australis* populations, while, they were negatively correlated with soil NO_3_^–^N in the same soil layer. Among them, seed germination rates and germination speeds had the greatest correlation with soil salinity of original habitats of *P. australis* populations, which was consistent with the results of seed germination experiment with salt addition treatment (**Figure 4a**). When seeds stored under the room temperature, seed germination rates were positively correlated with soil N/P and soil microbial biomass carbon in 10–20 cm layer, and negatively correlated with soil TP content of original habitats of *P. australis* populations. Seed germination potentials were positively correlated with soil pH value, microbial biomass carbon content in 0–10 cm layer, and AVIP in 10–20 cm layer soil, and negatively correlated with soil NH_4_^+^-N in 10–20 cm layer of original habitats of *P. australis* populations. Seed germination indexes and germination rates were positively correlated with soil AVIP, salinity and soil moisture in 10–20 cm layer, and negatively correlated with soil NO_3_^–^N of original habitats of *P. australis* populations (**Figure 4b**).

**Figure 5:**
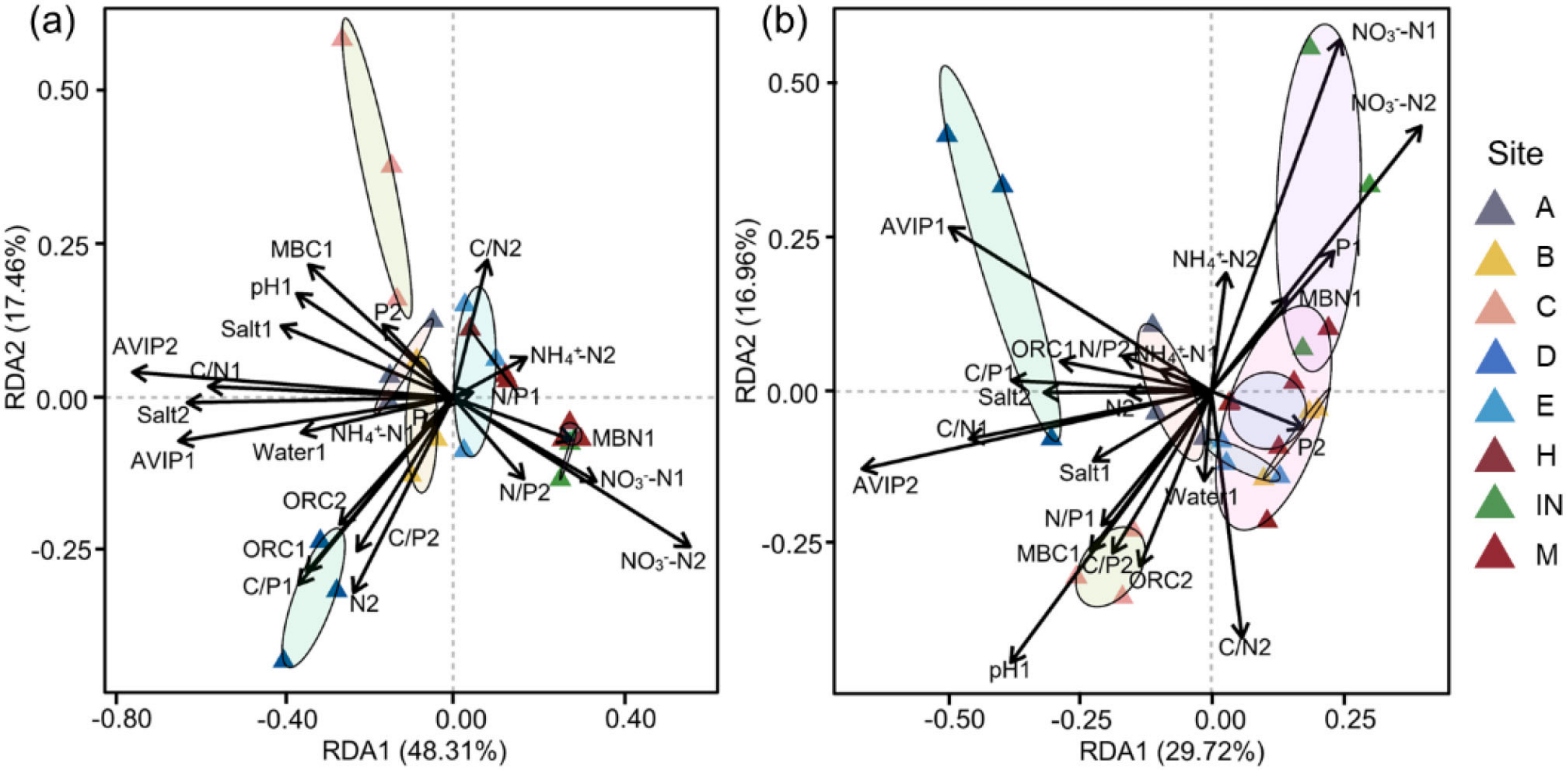
Redundancy analysis of soil physicochemical properties of the original population habitats and germination rates of seeds collected from the populations at different tidal flats **(a)** stored at 4°C and **(b)** stored at room temperature.

In order to further explore the effects of interaction between germination parameters of seeds under different seed storage temperatures and the key soil physicochemical factors of original habitats of *P. australis* populations, RDA was conducted, which showed that germination rates of seeds collected from the populations at different tidal flats and stored under 4 °C were significantly different. Taking the germination rates of seeds stored at 4°C as response variables, and soil salinity, NO_3_^–^N, NH_4_^+^-N, TN, TP, organic carbon content, moisture content, pH, AVIP, MBC and MBN of original habitats of *P. australis* populations as the explanatory variables, RDA was further conducted, which showed that the first and second ranking axes of RDA explained 65.77% of the impact of 0–10 cm layer soil physicochemical properties on the variation of seed germination performance. In the RDA ranking diagram, AVIP, C/N of 0–10 cm soil layer, C/P of 0–10 cm layer, and NO_3_^–^N of 10–20 cm layer of original habitats of *P. australis* populations were most closely related to the first axis. In addition, soil salinity, pH at 0–10 cm soil layer, and soil water content in 0–10 cm layer, MBN and NO_3_^–^N were also strongly correlated with the first principal component. In the soil physicochemical properties, soil TN had the greatest correlation with the second principal component, which followed by soil C/N. Cluster analysis showed that the germination rates of seeds collected from the populations at the middle and high tide flats had a strong correlation with NO_3_^–^N, C/N, N/P and NH_4_^+^-N of 0–10 cm and 10–20 cm soil layers, and the germination rates of seeds collected from the populations at the low tidal flat were correlated closely with other soil physicochemical properties of original habitats of *P. australis* populations (**Figure 5a**).

The relationship between soil physicochemical properties of original habitats of *P. australis* populations and the germination rates of seeds collected from the populations at different tidal flats and stored at the room temperature was also analyzed through RDA, which showed that total RDA results explained 46.68% of the impact of soil physicochemical properties on the seed germination performance, and the first axis included most of the effective results (**Figure 5b**). Result showed that AVIP, C/N, C/P and NO_3_^–^N content of 0–10 cm soil layer, were most closely related to the first axis. In addition, soil pH, TP in 0–10 cm soil layer, and soil C/N, NH_4_^+^-N in 10–20 cm soil layer were also strongly correlated with the second axis. Cluster analysis showed that germination parameters of seeds collected from the populations at the middle and high tidal flats, respectively, had strong correlation with soil NO_3_^–^N, TP, C/N and NH_4_^+^-N in 10–20 cm soil layer. The germination rates of seeds collected from the populations at the low tidal flat were significantly correlated with other soil physicochemical properties (**Figure 5b**). The RDA analysis results on the relationships between germination rates of seeds collected from the populations at different tidal flats and stored at different temperatures and the soil physicochemical properties showed that seed germination rates had the same trend of correlation with soil NO_3_^–^N content, AVIP, pH and C/N of the soil layers of original habitats of *P. australis* populations (**Figure 5**).

### The effects of soil physicochemical factors of original habitats of *P. australis* populations on the differences in seed germination parameters

**Figure 6:**
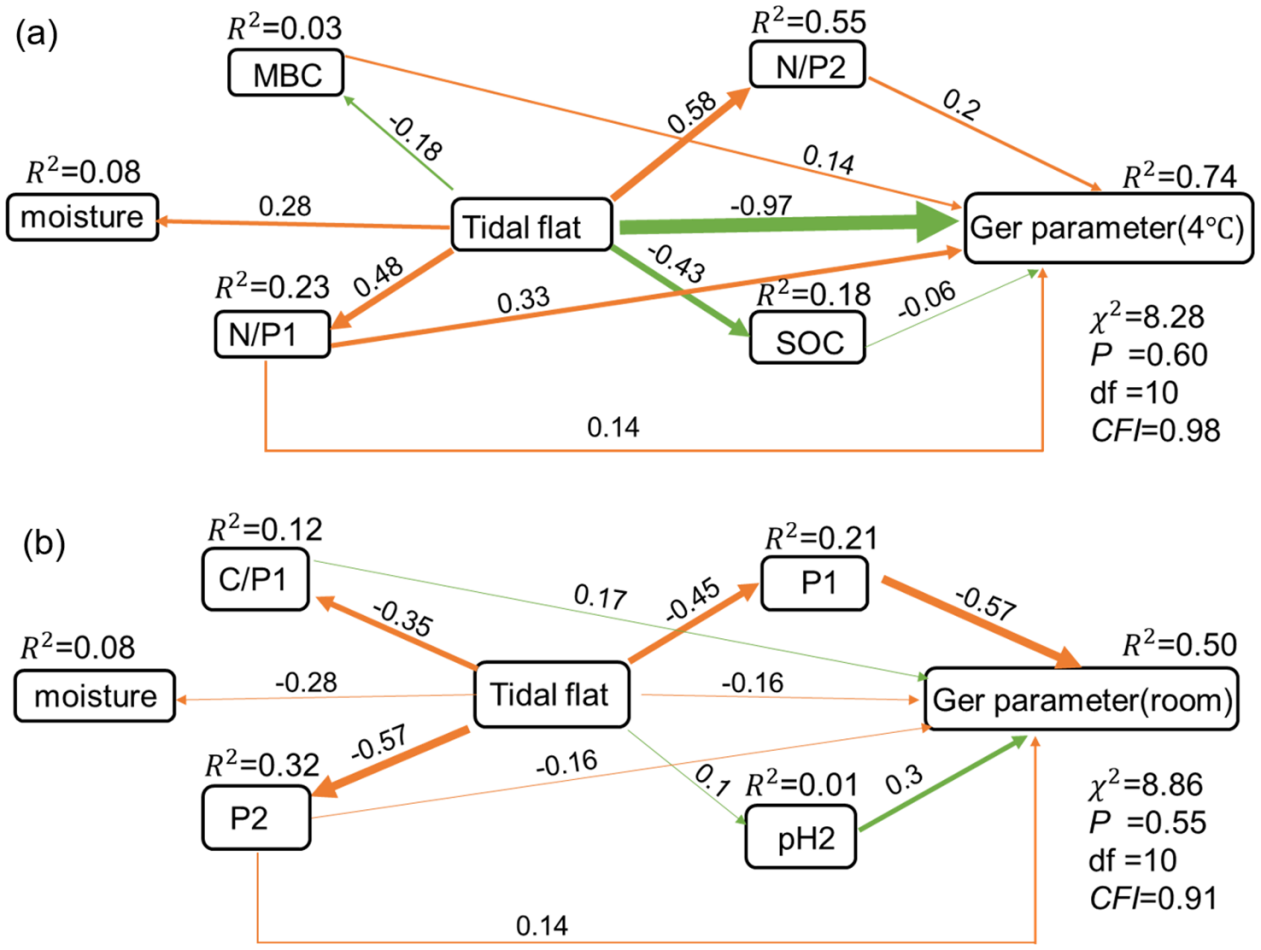
Structural equation model (SEM) for the germination parameters of seeds collected from the populations at different tidal flats and stored under different storage temperatures, and the soil physicochemical properties of the original population habitats. **(a)** 4°C storge condition, **(b)** room temperature storge condition.

The germination rates, germination potentials, germination indexes and germination speeds measured in this study were represented as a germination parameter of the germination strategy to construct a structural equation model (SEM). The SEM results showed that under the seed storage condition at 4°C, soil water, N/P1, N/P2 and soil microbial biomass carbon content of original habitats of *P. australis* populations affected seed germination. The path coefficient between soil moisture, soil N/P1, N/P2, MBC and seed germination was 0.14, 0.33, 0.2 and 0.14, respectively (**Figure 6a**). The tide level directly reduced the soil organic carbon content of original habitats of *P. australis* populations, and then indirectly led to the decline of the germination rates of seeds stored at 4°C (**Figure 6a**). The tidal level promoted the increase of soil moisture content, nitrogen and phosphorus ratio of original habitats of *P. australis* populations, which indirectly led to the increase of seed germination rates under the same seed storage conditions (**Figure 6a**). Soil pH of original habitats of *P. australis* populations was the most important factor affecting seed germination, when seeds were stored at room temperature, and the path coefficients between soil water content, C/P1, pH2 and seed germination was 0.14, 0.17 and 0.3, respectively (**Figure 6b**). The path coefficient between tidal flats of original habitats of *P. australis* populations and germination of seeds stored at room temperature was -0.16, while the path coefficient between tidal flats and germination of seeds stored at 4°C was -0.97. The tide level directly increased soil pH, which indirectly led to increase in germination rates of seeds collected from the populations at different tidal flats and stored at room temperature, while, the decrease of soil moisture content and phosphorus content among different tidal flats indirectly lead to the decrease of germination rates of seeds under the same storage conditions (**Figure 6b**).

## Discussion

### Response of seed germination to salinity in gradient tidal flat *P. australis* population

The two essential and critical developmental stages in the life cycle of an annual plant were regarded as flowering and seed germination (Ehrlén et al., 2015). Seed germination is usually the first step in the establishment of a plant population. Numerous studies demonstrated the effects of environmental factors such as temperature, salinity, soil moisture and other soil factors on seed germination (Gul et al., 2013; Fernandez-Torquemada and Sanchez-Lizaso, 2013; Gorai et al., 2006; Kettenring and Whigham, 2009). Soil salinity was found to be one of the vital factors directly or indirectly affecting the germination of seeds of *P. australis* populations along the Dongtan salt marsh wetland (**Figure 2**). Generally, the germination rates and germination potentials of *P. australis* seeds decreased with the increase of soil salinity (Wijte and Gallagher, 1996; Zhao et al., 2021). Seed germination rates of *P. australis* populations in the Yellow River Delta were also found to be inhibited seriously under the condition of high salt (2% - 3%) (Yu et al., 2012). Our research revealed that *P. australis* seed germination rates topped 80% under 0 salinity conditions (**Figure 2**). However, when exposed to 2% salinity, the germination rates of *P. australis* seeds from diverse tidal flats decreased to varying degrees, and some even failed to germinate. In addition, seeds at 2% salinity from middle tidal flats failed to germinate compared to those from the low tidal flat (80.9% ± 0.06%). These suggested that *P. australis* seeds from the low tidal flat possessed a higher tolerance and adaptability to salinity. Adaptation to a saline habitat that is the low-tidal zone is a likely explanation for our observation (Guan et al., 2016; Haraguchi, 2014). Under the two storage temperature conditions, the germination rate, germination index, germination potential and germination speed of L and L(IN) were all different. This might be due to the construction of the dike, which led to the reduction of hydrological connectivity, thus resulting in the differentiation of germination strategies between these two populations. Consequently, *P. australis* population could potentially establish and expand gradually based on the seeds of pioneer *P. australis* genets in low tidal habitats with relatively high soil salinity.

The germination rates of *P. australis* seeds in the Yangtze River Delta were slightly affected when salinity reached 1%. In contrast, the same salinity level led to a decreased germination rate for *P. australis* seeds collected from inland regions (Yu et al., 2012). Researchers studied the salt tolerance of *P. australis* seeds in salt marsh wetlands and inland estuaries, revealing the same pattern that the germination rates of *P. australis* seeds collected from the salt marsh wetland population were higher than those of seeds collected from the estuarine population (Haraguchi, 2014). The two-way ANOVAs testing the effects of salinity, the tidal flats and their interactions on the germination indexes of *P*. australis, results showed that salinity was the main factor affecting germination rate (Gt) and germination speed (Gs), while the tidal flat was the main factor affecting germination index (GI) and germination potential (GP) (**Table 1**). It is well known that salinity is negatively correlated with Gt and GI, low concentrations of NaCl induce seed dormancy, and high concentrations of NaCl induce osmotic stress and ionic toxicity (accumulation of Na^+^ and Cl^-^) and inhibit seed germination (Rajabi Dehnavi et al., 2020). In fact, for seeds in a salinity environment, due to the low osmotic potential of the germination medium, salinity can change the imbibition process of seeds to water, and water diffused from low salinity to high salinity, delaying the absorption of water by seeds, thus reducing germination (Farooq et al., 2015). Under salt stress, Na^+^ and Cl^−^ may be absorbed by seeds, and the toxic effect of NaCl may occur, thus slowing down the germination index and germination speed (Ashrafi and Razmjoo, 2015). The high germination rates, fast germination speeds and high germination vitality were found in *P. australis* seeds collected from the low tidal flat population growing in habitats with high soil salinity and high tide disturbance frequency (Baldwin et al., 2010; Briea, 2006). Our study also has similar results, low tidal flat *P. australis* seed Gt is higher, Gs is faster and GP is better than other tidal flats. **Table 1** shows that there are significant differences in germination parameters (Gt, Gs, GP, and GI) between different tide levels. As shown in **Figure 4**, different germination parameters are significantly correlated with some factors in soil physicochemical properties, which indicates that soil sediments and nutrient availability directly or indirectly affect seed germination traits of the reed populations. Such seed germination strategy may help accelerate the recruitment and establishment of *P. australis* populations at the low tidal flat of the Dongtan salt marsh wetland. Greenberg et al. (2001) revealed that most of the time plant seeds tended to adopt a “sit or wait” germination strategy, i.e., seeds sitting in their habitats and waiting for favorable opportunities for germination caused by environmental disturbances. However, the germination rates, speeds, and vigor of *P. australis* seeds from middle and high tide levels were inferior to those from low tidal flat populations. This suggested that the underground seeds of *P. australis* populations in middle and high tide levels might employ a “sit or wait” germination strategy, i.e., they could germinate when favorable opportunities arose. Along the Dongtan salt marsh wetland, the local adaptation of *P. australis* populations to the edaphic environmental conditions (especially the soil salinities) in the habitats shaped the sexual reproduction and seed germination strategies of *P. australis* populations as well as the seedling growth, showing the maternal effect in the local populations. In other words, the sexual progeny of *P. australis* populations at different tide levels in the Dongtan salt marsh wetland, apparently inherited the adaptive characteristics of the original ancestral populations, suggesting that when the sexual offspring of *P. australis* populations colonize the same habitats of their parental populations at different tide levels, they might perform well and maintain the long term structural and dynamic stability of the local populations.

### Effect of chilling environment on seed germination

70% of plant seeds have different types and degrees of dormancy, and plants need specific mechanisms to avoid adverse environmental conditions in nature, such as low temperature and dryness, and try to ensure that plants germinate in an environment suitable for seedling establishment (Afroze and O’Reilly, 2017; Baskin et al., 2003). Dormancy of gramineous seeds is a common phenomenon, and low temperatures in winter could efficiently break the dormancy of seeds of *P. australis*, suggesting the rejuvenation of seed germination vigor needs a short and proper cold treatment (Milberg, 1999; Li et al., 2013). The germination rates of *P. australis* seeds collected from the low tidal flat populations under 4°C storage condition for a while was significantly higher than those under the storage condition at room temperature (20 ± 1°C) for the same duration (**Figures 2**, **3**), indicating that different storage temperatures affected the germination vigor of *P. australis* seeds. The low-temperature storage condition (4°C) promoted the germination rates. Whereas, when *P. australis* seeds collected from the populations at the middle and high tidal flats were stored in the room temperature condition, the germination rates of seeds were improved, compared with those stored in 4°C condition (**Figure 3**), which implied that low temperature, as one of the environmental seize factors for seed germination, regulated the after ripening of seeds collected from the populations at the low tidal flats. Low temperature mainly showed slow germination speed and low germination rate (Akinnuoye and Modi, 2015). As a major environmental stress factor, low temperature can break the original metabolic balance of seeds, destroy the enzyme system, accumulate toxic substances, and thus affect the growth and development of plants (Greenwood and MacFarlane, 2006). Temperature is critical to plant growth and development, and many plants in temperate regions can remember past winter seasonal temperature drops and undergo changes in after ripening patterns to ensure the survival and/or reproductive success of offspring (Gao et al., 2022; He and Li, 2018). However, our findings demonstrated that low-temperature treatment enhanced the germination of *P. australis* seeds collected from low tidal flat populations in the Dongtan coastal salt marsh wetland in a subtropical climate zone. This improvement was notable despite the lack of the same effect on seeds from middle and high tide flats.

The Dongtan wetland (especially the frontier low tidal flat) exposes to frequent wind and sea tidal impact, whose standing water often freezes in winter whenever there is cold weather; thus, after long-term adaptation, the germination of seeds from the low tidal flat populations required moist chilling condition was quite understandable. The chilling environment near the coastal wetland, the metabolism of stored protein was changed by inducing ROS production and reducing the activity of protease in seed cells, thus affecting the seed germination rate (Shutov et al., 2003). Therefore, the seeds produced by the low tide flat *P. australis* population need after-ripening, breaking dormancy after low temperature environment, and successfully germinating in suitable condition. As for the fact that the germination of seeds collected from the middle and high tidal flats with less ecological stresses in the habitats no longer required after ripening might be attributed to the comprehensive impacts of multiple physiological and environmental factors. Our results showed that the contents of NH_4_-N and NO_3_-N in the soil were higher than those at the low tidal flat. The contents of Organic C, MBC and MBN in high tidal flat were higher than those in low tidal flat (**Table 2**). This means that the middle and high tide zone environment can provide a relatively stable soil sediment and availability of plant nutrients compared to the low tide zone environment with high frequency tidal disturbance. Meanwhile, previous studies have found that the soil structure of the middle and high tide zone of Dongtan salt marsh was sandy or silty soil, while the soil quality of the low tide zone was sandy clay (Liu et al., 2021; Jia et al., 2022). Sandy soil and sandy loam with large pores and low organic matter content are suitable substrates for the germination of *Jatropha curcas* seeds. The germination rate and germination speed of seeds with clay texture were lower (Valdés-Rodríguez et al., 2013). The soil texture of *P. australis* population at different tide levels affected the proportion of soil pore water and the content of organic matter, which would change the germination of seeds and the growth and development of plants. The seeds collected from low tidal gradients germinate better when stored at low temperatures, while seeds from middle and high tidal gradients germinate better when stored at room temperature. This phenomenon is one of the ecological drivers for seed germination in this species, as at a low tidal gradient, seeds may experience low and fluctuating temperatures due to a low water table. In contrast, at a middle and high tidal gradient, seeds may experience warmer and constant temperatures due to higher water table (Xiao et al., 2009).

### Response of early life history of seeds to habitat

Seed germination is the first stage of plant life, but the abiotic stress environment during seed germination can reduce seed germination by increasing seed quality degradation and reducing seed germination potential and vigor (Wang et al., 2024). The low temperature was often the key factor stimulating the seed germination vigor, while other conditions, such as suitable water, oxygen and nutrients, are still necessary (Lombardi et al., 2019). SEM results showed that germination of *P. australis* seeds stored at 4°C was affected by soil MBC, N/P ratio and soil water content in the original population habitats from which seeds were collected (**Figure 6**). Significant differences in soil pH, TN and organic carbon content in soil layers (*p*<0.05) were found among habitats from which *P. australis* seeds were collected, and the soil salinity, soil available nitrogen decreased with the increase of tidal flat, while other soil indexes showed no significant differences among the habitats (**Table 2**). The availability of certain elements is related to soil pH (Al-Wabel et al., 2018), thus, indirectly influencing the plant growing in the habitat. Soil pH is closely related to soil microbial diversity and indirectly affects soil MBC (Naz et al., 2022; Wan et al., 2021), while soil N/P affects the mineralization rate of soil, and further affect soil salinity synergistically with soil moisture (Miller and Geisseler, 2018), and indirectly affects seed germination rate (Feng et al., 2021).

As the mixed results of SEM analysis suggested, the relationship between soil properties, nutrients, and germination may be more complex. Former results also revealed the coupling effects of water, nitrogen and phosphorus in regulating the germination of wetland plant seeds (Zhao et al., 2019). Nitrate plays a role as a signaling molecule to mediate seed germination (Zhang et al., 2023; Li and Wang, 2015). Both endogenous and exogenous nitrate and phosphorus may affect seed germination (Song et al., 2016; Plassmann et al., 2008; Sims et al., 2012). The genetic diversity of *P. australis* patches in the Rhodes River Basin in the United States shows that almost all population patches are composed of multiple genotypes, and many patches are genetically different from other patches, which indicates that cross-fertilization is one of the sources of genetic diversity (McCormick et al., 2010). Therefore, the possibility of cross-fertilization and gene exchange between different populations may also affect the seed traits such as salt tolerance and germination rates across tidal zones (Lambertini et al., 2020). Except that, such as the water pollution, sediment and soil texture of tidal flats, structure, and availability of nutrients for plants also have direct or indirect influences on the seed germination strategy (Antonio Alburquerque et al., 2014; Bocchese et al., 2008; Peng et al., 2022; Satyanti et al., 2019; Wang et al., 1994). Chongming Dongtan Wetland is located in the lower reaches of the Yangtze River Delta, bearing the pressure of water pollution caused by population activities in the upper reaches, and the pollutants such as nitrogen, phosphorus and COD in the water can be removed by *P. australis* (Ho et al., 2024). Water pollution may cause significant differences in soil sediments between different tidal levels, and then affect the seed germination rate of different tidal flat populations. Wetland soil is the source, sink and transfer source of chemical pollutants. Anthropogenic activities (such as wetland reclamation, fertilization, and sewage discharge) have exacerbated changes in soil properties (such as SOM, pH, and Eh) of wetland soils/sediments (Bai et al., 2011). Absorption by the roots of *P. australis* can increase the residence time of these pollutants in the wetland, and then effectively reduce the diffusion of pollutants to the surrounding environment (Wang et al., 2015). In this study, *P.* australis seeds in coastal wetlands with high salinity showed a higher germination rate, which may be a trade-off in the face of tidal frequency and harsher environment.

## Conclusion

To consolidate the existing populations and facilitate their further expansion, as well as to prevent habitat fragmentation, it is recommended to more conservation of *P. australis* populations which is adaptive to the high salinity environment in the low-tide zone with high physical and biological pressure in Dongtan salt marsh. As for the *P. australis* populations in the middle and high tidal flats, proper cultivation and maintenance should be carried out in the early stages and the external interference and damage should be avoided, so as to help the populations successfully cross the environmental sieve in the early growth stages and complete life history. Furthermore, *P. australis* seeds derived from the different tidal flat populations should be used actively in restoring or reestablishing the original populations at the same habitat, respectively. However, it should be noted that due to the frequent erosion of tides, the dynamic changes of water salinity, environmental temperature and soil physicochemistry have complex effects on the germination of *P. australis* seeds in soil seed bank, and the germination strategy is also dynamically changing. We need to be very careful about extrapolating our laboratory results to natural ecosystems.

## Data Availability

The original contributions presented in the study are included in the article/**Supplementary Material**. Further inquiries can be directed to the corresponding authors.
