# Supplementary material for: Divergent germination strategies of Phragmites australis seeds for tidal flat gradient adaptation and the implications for coastal wetland restoration
Source: Front Plant Sci. 2025 Jul 29;16:1598379. doi: 10.3389/fpls.2025.1598379 (PMC12339510; doi:10.3389/fpls.2025.1598379)
Supplement: Supplementary file 1 [file SupplementaryFile1.docx]

**Divergent germination strategies of *Phragmites australis* seeds for tidal flat gradient adaptation and the implications for coastal wetland restoration**

Peng Jia^1, 2^, Dezhi Li^1,3,4,5^^[[1]](#footnote-1)^*, Caifen Yu^2^^[[2]](#footnote-2)^*, Jing Jia^1, 6^^[[3]](#footnote-3)^*, Jiangtao Wang^4^, Ying Wang^1^, Jing Chen^1^

1. School of Ecological and Environmental Science, East China Normal University, 500 Dongchuan Rd, Shanghai, 200241, China

2. National Marine Environmental Monitoring Center, Dalian, 116023, China

3. Shanghai Key Lab for Urban Ecological Processes and Eco-Restoration, 500 Dongchuan Rd, Shanghai, 200241, China

4. Institute of Eco-Chongming (IEC), 20 Cuiniao Rd, Chenjia Zhen, Chongming, Shanghai, 202162, China

5. Technology Innovation Center for Land Spatial Eco-restoration in Metropolitan Area, Ministry of Natural Resources, 3663 N. Zhongshan Road, Shanghai, 200062, China

6. Department of Ecology and Resources Engineering, Hetao College, Bayannur 015000, China

**Figure:**

**Figure S1** 100-grain weight of *Phragmites australis* seeds in different sample plot (ns: not significant).


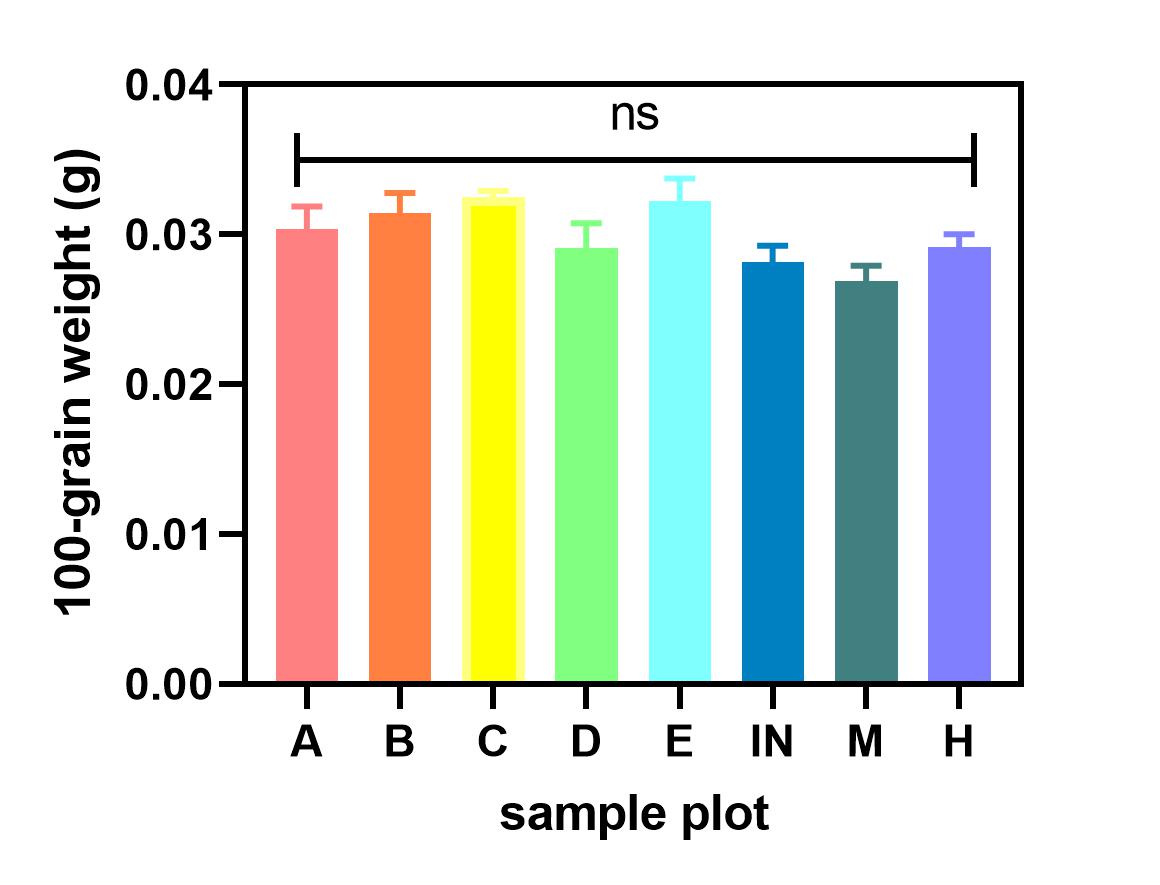


**Tables:**

**Table S1** Effects of salt stress on germination of reed seeds stored at 4℃ from different tidal levels. (Different lowercase letters indicate significant differences between different tidal levels groups, ns, no significant; P<0.05.)

**Table S2** Effects of salt stress on germination of reed seeds stored at room temperature from different tidal levels.

**Table S1**

|  | **NaCl concentration /‰** | **Tidal level** | | | |
| --- | --- | --- | --- | --- | --- |
|  |  | **L** | **M** | **H** | **IN** |
| Germination index | 0 | 0.5083±0.1912a | 0.385±0.14743a | 0.1043±0.0340bc | 0.1498±0.0227ab |
|  | 5 | 0.3243±0.1290a | 0.2604±0.1386a | 0.062±0.0149b | 0.0642±0.0234b |
|  | 10 | 0.1251±0.085a | 0.1444±0.0692a | 0.0116±0.0027ab | 0.0233±0.0050ab |
|  | 15 | 0.0651±0.0516ns | 0.0646±0.0081ns | 0.0074±0.0128ns | 0.0267±0.0095ns |
|  | 20 | 0.0187±0.0266ns | 0 | 0 | 0.0051±0.0087ns |
| Germination speed | 0 | 10.4946±1.7814a | 7.3596±0.8283b | 1.4922±0.2487c | 3.1768±0.6286b |
|  | 5 | 6.8445±2.1008a | 2.6244±0.3975b | 1.2005±0.1501b | 1.0013±0.3092b |
|  | 10 | 1.8555±1.2386a | 1.3737±0.1703a | 0.1212±0.0461a | 0.3935±0.0335ab |
|  | 15 | 0.5846±0.2690a | 0.3485±0.0850a | 0.037±0.0641b | 0.1337±0.0089a |
|  | 20 | 0.2031±0.03265a | 0 | 0 | 0.0303±0.0524b |
| Germination  potential | 0 | 0.519±0.0586a | 0.3067± 0.0339b | 0.0667± 0.0188c | 0.08±0.0163c |
|  | 5 | 0.3052±0.0457a | 0.0333± 0.0339b | 0.0411± 0.0340b | 0.033±0.0188b |
|  | 10 | 0.0526±0.0244ns | 0.0267± 0.0094ns | 0.0139± 0.0196ns | 0.013±0.0094ns |
|  | 15 | 0.0074±0.0055ns | 0 | 0 | 0 |
|  | 20 | 0.0011±0.001ns | 0 | 0 | 0 |

**Table S2**

|  | **NaCl concentration /‰** | **Tidal level** | | | |
| --- | --- | --- | --- | --- | --- |
|  |  | **L** | **M** | **H** | **IN** |
| Germination index | 0 | 0.5261±0.46417a | 0.2706±0.27499ab | 0.1411±0.06126ab | 0.1004±0.038ab |
|  | 5 | 0.2046±0.1629ns | 0.1121±0.07681ns | 0.0603±0.01154ns | 0.0989±0.10891ns |
|  | 10 | 0.0861±0.07252ns | 0.0086±0.00765ns | 0.0276±0.00372ns | 0.0302±0.03684ns |
|  | 15 | 0.0477±0.03691ns | 0.0086±0.00765ns | 0.0503±0.03255ns | 0.0158±0.0034ns |
|  | 20 | 0.0169±0.02977 | 0.0022±0.00377 | 0 | 0.0183±0.02397 |
| Germination speed | 0 | 9.2541±1.99597a | 3.6019±1.53721b | 2.9136±0.73965b | 1.0873±0.41958b |
|  | 5 | 4.4165±0.55782ns | 1.566±0.66235ns | 1.3381±0.26515ns | 0.9376±0.52969ns |
|  | 10 | 1.2313±0.51601a | 0.703±0.06721b | 0.5408±0.29203b | 0.2108±0.17783b |
|  | 15 | 0.3818±0.05245ns | 0.0703±0.06721ns | 0.1629±0.06896ns | 0.0988±0.04209ns |
|  | 20 | 0.2019±0.14018ns | 0.0196±0.03396ns | 0 | 0.1177±0.14651ns |
| Germination  potential | 0 | 0.1866±0.0741a | 0.1667± 0.1226a | 0.12±0.1177a | 0.0533± 0.0188b |
|  | 5 | 0.048±0.0231b | 0.083±0.090a | 0.0434± 0.0354b | 0.0266± 0.0188b |
|  | 10 | 0.0106±0.046ns | 0.0278± 0.0196ns | 0.0289± 0.0204ns | 0.0133± 0.0188ns |
|  | 15 | 0.0044±0.005ns | 0 | 0.0145± 0.0204ns | 0 |
|  | 20 | 0.0017±0.0025ns | 0 | 0 | 0 |

1. * Corresponding author: Dezhi Li. E-mail: dzli@des.ecnu.edu.cn [↑](#footnote-ref-1)
2. * Corresponding author: Caifen Yu. E-mail: yucaifen25@163.com [↑](#footnote-ref-2)
3. * Corresponding author: Jing Jia. E-mail: 52203903007@ stu.ecnu.edu.cn [↑](#footnote-ref-3)
